# Viral Vector-Mediated Antisense Therapy for Genetic Diseases

**DOI:** 10.3390/genes8020051

**Published:** 2017-01-26

**Authors:** Marine Imbert, Gabriella Dias-Florencio, Aurélie Goyenvalle

**Affiliations:** INSERM U1179, Université de Versailles St-Quentin en Yvelines and Université Paris Saclay, 2 Avenue de la Source de la Bièvre, 78180 Montigny-le-Bretonneux, France; marine.imbert2@uvsq.fr (M.I.); gabriella.dias-florencio-leite@uvsq.fr (G.D.-F.)

**Keywords:** antisense therapy, snRNA, viral vectors, splice-switching approaches

## Abstract

RNA plays complex roles in normal health and disease and is becoming an important target for therapeutic intervention; accordingly, therapeutic strategies that modulate RNA function have gained great interest over the past decade. Antisense oligonucleotides (AOs) are perhaps the most promising strategy to modulate RNA expression through a variety of post binding events such as gene silencing through degradative or non-degradative mechanisms, or splicing modulation which has recently demonstrated promising results. However, AO technology still faces issues like poor cellular-uptake, low efficacy in target tissues and relatively rapid clearance from the circulation which means repeated injections are essential to complete therapeutic efficacy. To overcome these limitations, viral vectors encoding small nuclear RNAs have been engineered to shuttle antisense sequences into cells, allowing appropriate subcellular localization with pre-mRNAs and permanent correction. In this review, we outline the different strategies for antisense therapy mediated by viral vectors and provide examples of each approach. We also address the advantages and limitations of viral vector use, with an emphasis on their clinical application.

## 1. Introduction

The use of antisense therapy to target RNA offers an effective approach for the treatment of genetic diseases. Antisense oligonucleotides (AOs) are short single-stranded nucleotide sequences, able to bind pre-mRNA and mRNA to modulate gene expression. Pioneered for therapeutic use by Stephenson and Zamecnik in 1978 [1], oligodeoxyribonucleotides were first used to prevent viral replication through inhibiting the translation of viral proteins. However, since this initial research, later attempts have demonstrated that short oligonucleotides are readily degraded by nucleases thereby reducing their efficacy. In order to avoid degradation, AOs may be chemically modified e.g., by phosphorothioate linkage, 2′-*O*-methyl or 2′-*O*-methoxyethyl. Several modified AOs have demonstrated encouraging outcomes for the treatment of different inherited diseases, such as muscular dystrophies or spinal muscular atrophy. However, synthetic antisense oligonucleotides present several disadvantages including limited cellular-uptake, efficacy and specificity for targeted delivery, and rapid clearance from the system following intravenous (IV) injections. These characteristics drive the need for repeated administrations in order to achieve a therapeutic response, with the negative consequence of accumulation in tissues and associated-toxicity. In order to achieve a long-lasting therapeutic effect without repeated injections, a vectorized system is required. Indeed, it is possible to modify small nuclear RNAs (snRNAs) with specific antisense sequences, and introduce them into viral vectors to get a long-term correction. In order to achieve effective intracellular expression of the antisense sequence, three main parameters must be considered: tissue tropism, in vivo stability, and subcellular localization of the molecular tools with the target pre-mRNA, all of which may be addressed using a snRNA-based approach.

In this review, we shall discuss the emergence of the snRNA system to shuttle antisense sequences with the use of different viral vectors, and provide examples of therapeutic applications for genetic diseases. Then we summarize the strengths and weaknesses of this strategy compared with AOs and other gene therapy approaches.

## 2. The snRNA System

### The Development of the snRNA Shuttle System

**Figure 1:**
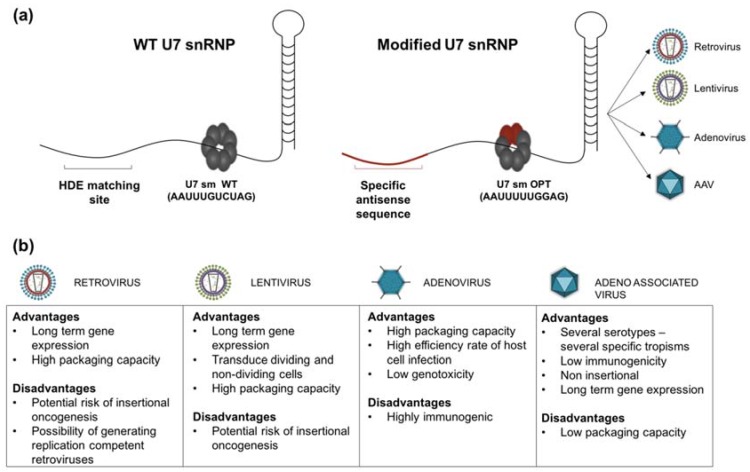
(**a**) Structure of U7 small nuclear ribonucleoproteins (snRNPs). snRNPs are the complexes formed by specific snRNA and the protein complex called Sm core, composed of seven Sm or Lsm (Sm-like) proteins encircled around the snRNA Sm binding site. The hairpin at the 3′ end plays a crucial role in snRNP stabilisation. The exposed 5′ end of the U7 snRNA is complementary of the histone downstream element (HDE) found in histone pre-mRNA, and is central in histone RNA 3′ end processing (WT U7 snRNP). The Sm core of the U7 snRNP consists of seven proteins: Lsm10, Lsm11, B/B’ (alternative splicing products), D3, E, F and G. For therapeutic use, U7 snRNA is genetically modified to modulate the splicing in different diseases. The modified U7 snRNP carries a different antisense sequence (specific to the target gene) and the specific U7 Sm binding site is replaced by the consensus sequence derived from the spliceosomal snRNPs (U7 Sm OPT); this modification results in more efficient accumulation of the U7 snRNP in the nucleus and the inability to cleave the histone pre-mRNA target due to the replacement of Lsm 10 and 11 by D1 and D2 Sm proteins. The modified U7 snRNA can also be equipped with a 5′ tail carrying an exonic splicing enhancer (ESE) or silencer (ESS) sequence (bifunctional), able to bind specific splicing enhancer/silencer (SE/SS) factors optimizing the effect of exon-skipping or exon reinclusion; (**b**) Advantages and inconvenient of main viral vectors types.

snRNAs are involved in the processing of pre-mRNA and are associated with specific proteins, called Sm core to form a complex of small nuclear ribonucleoproteins (snRNPs) (Figure 1a). U7 small nuclear RNA (U7 snRNA) is a component of the small nuclear ribonucleoprotein complex (U7 snRNP), normally involved in histone pre-mRNA 3′ end processing, through a complementary sequence to the histone downstream element (HDE). U7 snRNA can be used as a tool for pre-mRNA splicing modulation by modifying the binding site for Sm/Lsm (Sm-like) proteins [2]. Schumperli and colleagues showed that converting the Sm binding site of U7 snRNA (U7 Sm WT) into the consensus sequence derived from spliceosomal snRNPs (U7 SmOPT) resulted in the assembly of the Sm proteins D1 and D2 instead of the usual Lsm10 and Lsm11. This modification leads to the accumulation of U7 Sm OPT snRNPs in the nucleus and prevents the action of this complex on histone RNA processing [3,4]. Based on these observations, it has been suggested that modified U7 snRNPs carrying antisense sequences complementary to specific splicing sites could be suited for the manipulation of targeted genes. The antisense sequence carried by a snRNP will naturally accumulate in the nucleus where splicing occurs, and will be protected from degradation.

Similar studies have also investigated the use of modified U1 snRNA [5] and U6 snRNA [6,7] to target nuclear splicing sites. U1 and U6 are part of the spliceosome, along with U2, U4, U5 and several proteins [8]. Each snRNAs, except U6, is forming a complex with Sm core proteins to form a snRNP. The attractiveness of U1 as a therapeutic target is due to the fact that U1 snRNA expression is 6-fold higher than U7 snRNA expression per gene copy and because of its central role in splicing. Research however suggests that targeting U1 snRNA over the other snRNA constructs does not result in superior efficacy, perhaps due to the inability of the modified U1 to compete with the largely expressed wild-type U1 for specific biding sites [2,5].

The U7 smOPT snRNP technology was first tested in an in vitro model of β-thalassemia. This disease is caused by several mutations in the *HBB* gene, that codes for the β-globin protein. The model used in this study is a HeLa cell line carrying a T/G substitution in the position IVS2-705 of the human β-globin gene, that leads to abnormal splicing and β-globin deficiency. Results indicated that the use of a U7 snRNA carrying an antisense sequence targeting this mutation corrected more than 50% of aberrant splicing and subsequent re-expression of full-length β-globin protein [9]. Since this development, U7 smOPT snRNPs have been successfully exploited to correct mutations in a number of inherited diseases, including Duchenne muscular dystrophy (DMD) and spinal muscular atrophy (SMA) which are detailed below.

## 3. Therapeutic Applications

### 3.1. Splicing Modulation

**Figure 2:**
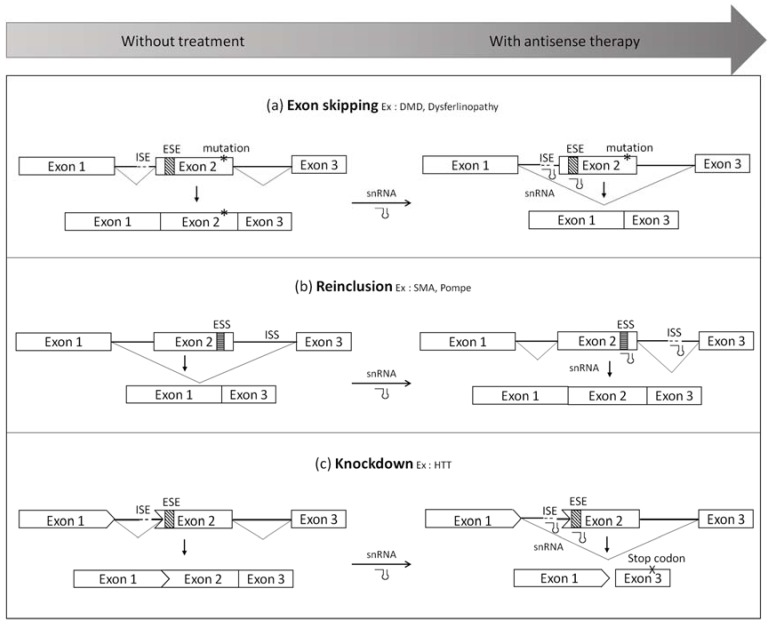
Splicing modulation mediated by small nuclear RNAs (snRNA) system (**a**) Exon-skipping. This approach consists in using modified snRNA to hide important splice sites such as the acceptor/donor splice sites or exonic splicing enhancers (ESE) in order to skip the mutated exon. It leads to a truncated protein, which is still functional; (**b**) Exon reinclusion. This strategy allows the inclusion of an exon by targeting silencer of splicing located in exons (ESS) or in introns (ISS); (**c**) Knockdown. snRNA can also be used to skip an exon which disrupts the open reading frame in order to create a premature stop codon. This abnormal transcript will be degraded, which will silence the gene expression. DMD, Duchenne muscular dystrophy; SMA, spinal muscular atrophy; HTT, huntingtin.

Antisense sequences can be employed to modulate splicing signals (Figure 2). For instance, they can be used to induce either exon-skipping or exon inclusion, or even to disrupt the open reading frame for gene knockdown. The therapeutic application of snRNAs-mediated splicing modulation will be discussed here using diseases such as DMD, SMA and Pompe disease as examples.

#### 3.1.1. Exon-Skipping

●  Duchenne muscular dystrophy

DMD is an X-linked recessive form of muscular dystrophy that affects 1 in 3600 boys. This disease is caused by mutations (deletions, duplications, insertions and point mutations) in the DMD gene which mostly disrupt the open reading frame and give rise to non-functional protein. DMD patients display total lack of dystrophin leading to progressive muscle degeneration and premature death. Interestingly, Becker muscular dystrophy (BMD), which is also due to mutations in the dystrophin gene, results in a less severe phenotype. In this case, BMD patients have a truncated dystrophin, internally-deleted but still functional. In contrast with DMD mutations, BMD deletions do not disrupt the open reading frame [10]. One of the most promising therapeutic approaches for DMD is to convert an out-of-frame transcript into an in-frame transcript which can be accomplished with antisense sequences that mask key splicing sites (Figure 2a). In 1996, Pramono and colleagues reported the first dystrophin exon-skipping in lymphoblastoid cells using antisense oligodeoxynucleotide [11]. Following this encouraging result, several in vivo studies provided pre-clinical evidence for the therapeutic potential of an antisense strategy for DMD in different animal models. One model in particular, the *mdx* mouse (carrying a nonsense mutation in exon 23), is being widely used to test the efficacy of the AO approach using various oligonucleotide chemistries such as 2′*O*Me, phosphorodiamidate morpholino oligomers (PMO), locked nucleic acid (LNA) or peptide nucleic acid (PNA) [12,13]. Two of these chemistries have been evaluated in clinical studies and demonstrated encouraging results (drisapersen [14,15,16,17,18,19] and eteplirsen [19,20,21,22,23]). However, although dystrophin could be restored at low levels using these synthetic naked oligonucleotide compounds in DMD patients, these studies have failed to show a marked clinical benefit, and while the US Food and Drug Administration (FDA) has surprisingly granted accelerated approval to eteplirsen (a PMO-AO targeting exon 51), additional clinical trials have been requested to confirm the drug’s clinical benefit which has not yet been demonstrated. Therefore, there is still a critical need to improve current antisense tools and their delivery which is still very limited. This is particularly important for DMD where all muscles included diaphragm and heart, need to be targeted. Furthermore, repeated injections of AOs are required to maintain splice-switching effects, leading to accumulation in tissues such as kidney and liver in particular and subsequent potential long-term toxicities [24].

To overcome these issues several groups have attempted to achieve permanent in situ expression of antisense sequences using viral vectors to maintain therapeutic levels of dystrophin, which has been shown to reach levels of between 15% and 30% of meaningful improvement [25,26,27]. In 2002, De Angelis and colleagues investigated the use of different snRNAs as an antisense shuttle, based on the work done on *HBB* gene [9,28]. These snRNAs were selected because they are involved in splicing events in the nucleus and are consequently in the same subcellular localization as the target pre-mRNA. This study compared various chimeric snRNAs containing antisense sequences, transducing DMD-derived cells with recombinant retroviral particles which resulted in the efficient skipping of exon 51 and partial rescue of dystrophin synthesis in vitro with U1 snRNA and U7 snRNA. Interestingly, the highest level of exon-skip was obtained with a U7 snRNA vector carrying two antisense sequences, which was called “double target U7” [28]. This result was confirmed by Brun et al. whom have also shown that the double target construct is the most efficient [29]. These in vitro studies have demonstrated the feasibility of using snRNA system as a therapeutic tool for DMD and have encouraged further work on animal models. Goyenvalle and colleagues have constructed a “double target U7” masking two important sites of splicing in introns 22 and 23. This engineered U7 snRNA was injected in *mdx* mice muscles using adeno-associated-virus (AAV) vectors which induced persistent exon 23 skipping that resulted in rescue of dystrophin, and importantly muscle function [30]. Subsequently, the efficacy of AAV-U7 snRNA to induce exon-skipping was evaluated in a much more severe mouse model of DMD, the utrophin/dystrophin double-knockout (dKO), resulting in a remarkable increase of their lifespan [31]. Comparable works were reported by Denti and colleagues using AAV vectors expressing either U1 or U7 snRNAs with local muscle injection in *mdx* mice. The group demonstrated that U1 like U7 is also active in exon-skipping and suitable for expression of antisense molecules [32]. In another study, the same group demonstrated restoration of dystrophin expression in *mdx* mice after systemic injection of AAV-U1 snRNA [33] and the therapeutic benefit was still observed 74 weeks later [34]. Restoration of dystrophin expression in cardiac muscle has also been demonstrated in a large animal model, the golden retriever muscular dystrophy dog (GRMD), using percutaneous transendocardial delivery of AAV-U7 snRNA [35]. Additional studies from Garcia and Moullier have shown correction of the dystrophic phenotype and partial recovery of muscle strength after intramuscular (IM) and IV injections of AAV-U7 snRNA in GRMD dogs [36,37]. Altogether these results have advanced the feasibility of exon-skipping in different animal models, suggesting promising therapeutic outcomes based on AAV-snRNA. Consequently, different groups have tried to identify the most appropriate antisense sequences targeting the human dystrophin pre-mRNA that are able to induce the highest level of skip. Incitti and colleagues have achieved skipping of exon 51 in human cells carrying deletions of exons 48–50 or 45–50 using U1 snRNA [38]. Appreciating that DMD is caused by mutations at different gene loci and that 70% are located between exons 45 and 55, Goyenvalle et al. have designed eleven U7 snRNAs targeting these different human dystrophin exons. They have provided very encouraging results in DMD-patient myoblasts and demonstrated a multiple skipping of at least 3 exons with a single vector in a human DMD transgenic mice (hDMD) [39]. This group has also investigated the use of a ‘bifunctional U7snRNA’ carrying a tail harbouring binding sites for a splicing silencer (hnRNPA1) in addition to the antisense sequence [40].

Altogether, these data have shown the efficacy of AAV-U7 snRNA-mediated exon-skipping therapy for DMD, even if this strategy still faces obstacles to maintain the long-lasting effect for clinical applications (reviewed below in the AAV Section 4.3).

●  Dysferlinopathy

Exon-skipping approaches mediated by snRNAs have also been successfully applied for dysferlinopathy. Dysferlinopathy is an autosomal recessive disease covering a wide spectrum of phenotypes caused by different mutations in the *DYSF* gene which encodes dysferlin. Some of them are in-frame deletions of exons resulting in typical to severe phenotypes, which inform that these exons cannot be skipped without important deleterious impact. However Sinnreich and colleagues have reported a physiological exon 32 skip, associated to a very mild phenotype [41]. Since this exon appears at least partially dispensable Wein et al. have managed to induce a high efficiency of exon 32 skipping either with AOs or with Lentivirus-U7 snRNAs [42], which may be a potential therapy for the few patients carrying a mutation in exon 32. These results support the conclusion that this technology could be adapted for other diseases which might benefit from splicing modulation.

#### 3.1.2. Exon Inclusion

Antisense technology may also be used to induce re-inclusion of exons that are skipped due to a mutation as represented in Figure 2b.

●  Spinal muscular atrophy

SMA is a common fatal autosomal recessive disorder, characterised by degeneration of α-motoneurons in the anterior horn of the spinal cord leading to progressive muscle weakness, paralysis and often death. SMA is caused by mutations in the survival motor neuron 1 (*SMN1*) gene encoding for SMN protein [43]. The human genome harbors a nearly identical paralog of *SMN1* called *SMN2* which differs by few nucleotides, including a C-to-T transition. This divergence results in exclusion of exon 7 from the *SMN2* transcript which induces the prevalent production of a truncated protein. Only 15% of the *SMN2* mRNA leads to functional full-length SMN protein, which cannot compensate for the lack of *SMN1* [44,45]. Each SMA patient retains at least one *SMN2* copy and German researchers have found an inverse correlation between SMA severity and the number of copies of the *SMN2* gene [46]. These findings highlight the therapeutic potential of exon 7 reinclusion in *SMN2* mRNA. The splicing regulation of this exon has been studied to better understand the mechanism. It appears that exon 7 is a poorly defined exon, with different exonic splicing enhancers (ESE) and silencers (ESS) creating a fragile equilibrium [47,48]. Crucially, introns 6 and 7 also carry important sequences such as intronic splicing enhancers (ISE) and silencers (ISS) which strongly affect the exclusion or inclusion of exon 7 in SMN transcripts [49]. Consequently researchers have designed an AO directed toward the 3′ splice site of exon 8 demonstrating modulation of *SMN2* splicing with an increase of transcripts containing exon 7 [50]. Singh and colleagues have described an ISS, called ISS-N1, located downstream of the 5′ splice site in intron 7. They investigated the role of this element in exon 7 skipping with an antisense oligonucleotide targeting this sequence. They showed the restoration of exon 7 reinclusion and an increase of SMN protein in SMA patient-derived cells [51]. Subsequently, Hua et al. performed a large screening of both flanking introns using AOs that inhibit different ISS, discovering an optimal sequence that increased exon 7 inclusion in liver and kidney of human *SMN2* transgenic mice after systemic injection [52]. This sequence targeting the ISS-N1 has been studied further by several groups using different AO chemistry and reinclusion of exon 7 in SMN transcripts as well as an increase of the SMN protein could be shown in vitro and in vivo [53,54,55,56,57,58,59]. These promising results led to the recent clinical evaluation of this approach using a 2′-*O*-methoxyethyl (2′MOE) AO named nusinersen (marketed as Spinraza) in SMA patients. While treatment of SMA patients type 2 and 3 showed modest efficacy [59], results from a phase 3 trial in type I patients appear very encouraging with an improvement of motor skills [60]. Based on these data nusinersen was approved by the FDA in December 2016.

Although a mortality benefit seems likely the survival question remains, especially considering the mode of administration of nusinersen which has thus far been injected directly into the cerebrospinal fluid (CSF). This is of particular note since peripheral pathology has been demonstrated in SMA mice [61]. This indicates that peripheral SMN restoration is essential suggesting that combined central and peripheral delivery would be required for optimal treatment. Unfortunately most AOs injected intravenously cannot cross the blood-brain-barrier (except for the recently described tricyclo-DNA-AOs and some Peptide conjugated-PMO PPMOs [62,63], highlighting the need for viral vectors which offer a large panel of tropism depending on their serotypes (reviewed below in Section 4).

In order to investigate the vectorization and achieve long-term correction without AO accumulation in untargeted tissues, antisense sequences were introduced in the snRNA system. The first study using U7 snRNA targeting the intron 7/exon 8 junction of *SMN2* reported a considerable exon 7 re-inclusion and an increase of SMN protein in HeLa cells [64] and in SMA patient derived fibroblasts [65]. An Italian group developed several exon-specific U1 snRNAs (ExSpeU1) that correct aberrant splicing in coagulation factor IX, cystic fibrosis transmembrane conductance regulator (*CFTR*) and *SMN2* [66]. After transduction of cells from SMA-affected patients with lentiviral particles expressing SMN-specific ExSpeU1, they demonstrated an augmentation of exon 7 inclusion leading to an increase of SMN protein. Most importantly they injected self-complementary AAV9 (scAAV9) carrying this therapeutic cassette intraperitoneally in transgenic mice at postnatal day 1, and showed the correction of the splicing defect in brain, heart, kidney, liver and skeletal muscle [67].

Schümperli and colleagues have developed bifunctional U7 snRNA based on bifunctional antisense oligonucleotide strategy. In addition to the antisense sequence these AOs carry either an SR peptide (containing repeats of serine and arginine residues, involved in splicing mechanism), or an ESE (exonic splicing enhancer) [68,69,70]. Both types of bifunctional AOs demonstrated an increase of SMN expression and a phenotype enhancement in mice model of SMA after intracerebroventricular injections [71,72,73]. Using the same principle bifunctional U7 snRNA carries an antisense sequence that binds to exon 7 and a free splicing enhancer sequence that promotes the inclusion of this exon. These constructs induce a nearly complete inclusion of exon 7 in *SMN2* transcripts and an extended increase of SMN protein level in HeLa cells and in SMA type I patient fibroblasts [74]. Subsequently they introduced this bifunctional U7 construct into the most severe SMA mouse model via transgenesis using lentivirus (LV) and observed a 20-fold increase in survival times (median of 123 days for U7-SMA mice compared to 6.5 days for SMA mice), normal weight development and muscle performance [75]. Interestingly, SMA mice expressing the therapeutic U7 cassette did not present ultrastructural changes in diaphragm neuromuscular junctions [76]. However, using this transgenic approach the study did not reveal if the phenotype could still be reverted when the therapy is administered after the first phases of post-natal life. This question was addressed in a recent study where they injected scAAV9 vector particles containing four copies of the U7 cassette into the cerebral ventricle of newborn SMA mice, and induced a significant increase in survival and muscle functions. Surprisingly the U7 snRNA was also expressed in heart and liver which was likely responsible for enhancing rescue of the phenotype [77]. All of these studies suggest that the correction of *SMN2* mRNA splicing and the alleviation of the phenotype not only work in a transgenic procedure but also in a somatic gene therapy approach.

●  Pompe disease

Another example of exon inclusion worth mentioning is the approach applied to Pompe disease, (glycogen storage disease type II) which is an autosomal recessive metabolic disorder caused by mutations in the acid α-glucosidase gene (*GAA*). Pompe disease leads to an accumulation of glycogen in the lysosome which damages muscle and nerve cells. The most common splice mutation is a T>G transition located in intron 1 which causes the skipping of exon 2, encompassing the start codon located within this exon. This mutation may modify the secondary structure of the pre-mRNA, altering the correct removal of intron 1. Our group has used AOs which simply bind to the 5′ region of exon 2 in order to restore the secondary structure and allow normal splicing. The expression of a functional protein GAA has been rescued in skin fibroblasts derived from a Pompe patient with this mutation. Furthermore, we have shown inclusion of exon 2 at the GAA mRNA level after transduction with a lentiviral vector encoding an U7 snRNA targeting exon 2. In addition, the rescue of the GAA protein in treated cells has confirmed the potential of this U7 as a therapeutic tool for Pompe disease (Avril et al. manuscript in preparation).

#### 3.1.3. Gene Knockdown

snRNA systems can also be used for exon-skipping to disrupt the open reading frame, thus creating a premature stop codon and preventing protein synthesis (Figure 2c). Schümperli’s group first adapted this method as a potential therapy for human immunodeficiency virus (HIV) infection reducing the synthesis of cyclophilin A (CyPA) which is required for HIV multiplication. Two U7 snRNAs were engineered to target splice sites flanking exons 3 and 4 of CyPA and then transfected into HeLa cells. They showed that “double-target” constructs induce very efficient exon-skipping. The group then used lentiviral vectors to achieve stable cell transduction, demonstrating strong diminution of full length transcripts and CyPA protein in three different cell types [78]. Years later this approach was applied to Tat and Rev which are HIV regulatory proteins, confirming its use as a therapeutic tool for gene knockdown [79].

Apart from its use as a therapeutic tool the snRNA system may also be used to silence gene expression in order to further our understanding of encoded proteins. To learn about the function of Ca^2+^ channel subunit called α1S, Garcia’s group have engineered an U7 snRNA able to silence α1S expression disrupting the open reading frame by exon-skipping. This construct was cloned into an AAV vector then injected intramuscularly in mice, leading to long-lasting downregulation of α1S which was still reduced 6 months after injection [80]. This research allowed better understanding of the α1S subunit and highlighted the potential use of U7snRNA as a gene silencer, similar to the RNA interference technology.

### 3.2. Reduction of Toxicity Due to Triplet Expansion

The antisense approach has also been used to specifically target toxic transcripts containing abnormally high numbers of triplet repeats, as in the case of myotonic dystrophy. Myotonic dystrophy type I (DM1) is an autosomal dominant neuromuscular disease occurring due to the expansion of a CTG trinucleotide repeat in the 3′ untranslated region (3′UTR) of myotonic dystrophy protein kinase (DMPK) [81,82]. The mutant DMPK presents various lengths of CTG expansions ranging from 800 to 2000 repeats, whereas normal-sized alleles contain fewer than 37 repeats. Mutant transcripts are retained in the nucleus and form nuclear foci [83]. It has been shown that CUG repeats form RNA hairpins that bind different proteins such as muscleblind-like 1 (MBNL1) [84], which is a regulatory splicing factor. In fact, the sequestration of MBNL1 in the nucleus prevent its activity and trigger misregulation of alternative splicing of several genes, like ClC-1 which encodes for the main chloride channel in skeletal muscle [85,86,87]. Furling and colleagues have produced a retrovirus carrying antisense sequences complementary to CUG repeats and 110-bp region following the repeats to be specific of DMPK transcripts. They have shown a decrease of mutant DMPK transcripts after transduction of human DM1 myoblasts. Also the phenotype of these cells was restored after treatment, confirming the hypothesis that antisense will release MBNL1 previously sequestered by CUG hairpins [88].

Other studies have shown the therapeutic potential of AOs targeting the CUG repeats [89,90,91,92]. Using different AO chemical modifications and lengths, they all demonstrated silencing of mutant DMPK RNA expression and a normalizing effect on aberrant splicing in vitro and in vivo. Furling’s group has also engineered a U7 snRNA targeting the CUG repeats and reported specific degradation of pathogenic DMPK mRNA after lentiviral transduction of myoblasts. Of interest, the number of foci was reduced and the splicing abnormalities were improved with the U7-(CAG)15 [93], implying the therapeutic potential of an snRNA based approach.

Altogether these approaches demonstrate the therapeutic potential of an snRNA based system, largely due to the properties of the viral vectors by which they are introduced.

## 4. Advantages of Vectorized Systems

In order to play their therapeutic role antisense sequences must reach the nucleus, however, delivery of naked nucleic acids is a challenge given their extra-nuclear instability and poor cellular-uptake. The main advantage of the snRNA-based approach is that it may be vectorized in many different viruses, allowing better delivery of therapeutic sequences to target cells versus synthetic oligonucleotides for example. Viral vectors are considered as one of the most promising means of gene therapy delivery. During the last four decades, virus engineering techniques have been developed and upgraded in order to produce recombinant viruses. These modified viruses have no replicating or pathogenic characteristics but retain their ability to cross the cell and nuclear membranes. Modifications of the viral structure and choice of promoter allows greater specificity concerning the cell type or tissue targeted and the extent of gene expression.

When choosing a viral vector one must consider several parameters: The size of the construct, the nature and size of the tissue or organ targeted, the amount of gene expression needed to achieve therapeutic success and whether or not long-term gene expression is required to overcome the disease. The snRNA based approach was vectorized in several types of viral vectors and the advantages and disadvantages of each one will be discussed below.

### 4.1. Retrovirus/Lentivirus

The snRNA based approach can be used as a therapeutic tool or more simply as a molecular tool when vectorized in retroviral or lentiviral vectors, depending on the required characteristics of these viruses and the disease to be targeted.

From the *Retroviridae* family, gammaretrovirus (MLV—murine leukemia viruses) and LVs are single stranded RNA viruses. Retroviral vectors were the most common gene transfer system used in gene therapy research until a trial for patients with X-linked severe combined immunodeficiency resulted in four out of nine treated patients developing leukaemia [94]. Retroviruses present two major safety issues: (I) the potential risk of insertional oncogenesis and (II) the possibility of generating replication competent retroviruses (RCR). Lentivirus’ capacity to produce replication competent LVs (RCL) is much lower with current viral vector systems [95] and several generations of lentiviral vectors have been constructed to improve their safety and efficacy [96], but they still present a potential risk of insertional oncogenesis. These safety issues can be a major problem when considering in vivo treatments but the insertional capacity may be beneficial when specific and stable long-term expression of the transgene is needed to achieve therapeutic success. For these reasons this type of vector has mainly been used as a molecular tool for in vitro studies. For example, lentiviral vectors in combination with U7 snRNA system were used to show the proof of principle that the inclusion of *SMN2* exon 7 could reduce SMA symptoms [74,75]. Successful in vitro studies with using LV vectors were also conducted for DM1 [93], Central core disease [97], hematopoietic diseases [98] and acquired immune deficiency syndrome [78,79] suggesting a therapeutic potential of the LV-snRNA strategy for these particular diseases.

Even though safety issues limit the use of this type of vectors in clinical trials, they remain an excellent tool for diseases that can benefit from ex vivo treatments such as hematopoietic stem cell grafts or as a molecular tool to establish a proof-of-principle in vitro.

### 4.2. Adenovirus

Few studies have used adenoviral vectors to vectorize U7 snRNAs. From the *Adenoviridae* family, adenoviruses are among the first studied viruses for use in gene therapy [99]. These are non-enveloped viruses with double stranded DNA genomes of about 26–45 kb in an icosahedral capsid. For use in gene therapy adenoviruses must be attenuated to avoid the dissemination of genetically modified vectors. The ability to delete the entirety of the adenovirus genome enables substantial coding capacity (up to 30 kb) [100]. More than 100 serotypes of adenoviral vectors have been reported, and serotypes 2 and 5 are often used for the advantage of being capable of transducing both dividing and non-dividing cells [101].

In SMA for example, one gene therapy study used adenoviral vectors of serotype 5 carrying the *SMN2*-antisense U7 snRNA to promote inclusion of *SMN2* intron 7 and successfully restored full-length SMN [65]. Among other advantages of adenoviral vectors, they are simple to purify and achieve high yields with. Furthermore, these vectors are non-integrative to the host genome conferring low genotoxicity. However, due to the high immunogenicity of the capsid adenoviral vectors are not the best candidates for systemic applications.

### 4.3. Adeno-Associated Virus

Recombinant adeno-associated viruses (rAAV) are largely used for gene transfer in research, preclinical development and clinical trials and are considered today the best candidates for gene therapy strategies in neuromuscular diseases, due to several attractive characteristics discussed in this section. AAV from the *Parvoviridae* family are small non-enveloped viruses with single-stranded DNA. The size of AAV particles ranges between 25 and 30 nm and as such they have a low packaging capacity. The capsid is composed by three proteins: VP1, VP2 and VP3 (ratio 1:1:10) and modifications in VP proteins determine their different tissue tropism. These are non-integrative vectors, and therefore present a low risk of genomic insertions that might lead to tumourigenesis. They are able to efficiently transduce several tissue-types and display long-term efficacy in post-mitotic tissues which make them good candidates for numerous gene transfer applications. However, the number of AAV genomes in dividing cells decreases gradually [102] which renders AAV the best choice for the transduction of slowly dividing cells such as myocytes or cardiomyocytes. Clinical trials using rAAV as gene therapy vectors have yielded promising results and the first marketing approval, using a rAAV1 vector, was delivered by the European Union in October 2012 for the treatment of familial lipoprotein lipase deficiency [103].

One of the main advantages of AAV vectors is their effective delivery to various tissues following systemic injection, though this depends on the serotype used. For example, AAV1 is mainly used for its high efficacy in transducing skeletal muscle following IM injections when proof of principle is intended. Several studies have investigated the efficiency of modified U7 or U1 snRNA for DMD using the *mdx* and dKO model. As a proof of concept, U7 and U1snRNA were vectorised in an AAV1 to transduce local skeletal muscles. When IV administration is required to target all skeletal muscles AAV serotypes -6 and -9 are most suitable [104]. scAAV-9 was used in the dKO model not only because of its ability to transduce skeletal muscle, but cardiac muscle particularly efficiently [31]. Moreover the use of scAAV revealed an enhanced transduction capability when compared to conventional single-stranded AAV vectors (ssAAV), by circumventing the need to convert the single-stranded DNA genome into double-stranded DNA prior to expression [105].

Regarding delivery to the CNS several AAV serotypes are capable of transducing CNS cells when injected directly in the CSF (-1, -2, -4, -5, -7, -8, -9 and rh10), however only AAV serotypes -1, -8, -9 and rh10 have demonstrated the ability to transduce CNS cells following IV injections [106,107].

These properties are particularly useful for the treatment of diseases like SMA where both the peripheral and central nervous systems require targeting. Comparison between AAV1 and scAAV9 revealed a greater transduction potential of the later to transduce motor neurons following IV injections [108]. This finding was confirmed in neonatal [108,109,110] and adult mice [108,111], as well as cats [108] and non-human primates [111,112]. Further comparison of AAV9 and AAVrh10 revealed that despite both serotypes being able to transduce CNS cells, at lower doses AAVrh10 is superior especially in the brainstem and cerebellum, which are important targets for the treatment of SMA type 1 [106].

A major disadvantage of AAV vectors for their use in gene therapy is their low packaging capacity, though this is not an issue for a snRNA-based approach given the small cassette size. This represents one of the main advantages of this strategy, since several snRNA cassettes may be inserted into a single AAV in order to induce double-skipping or even multi-skipping. One study reported the efficacy of eleven U7 snRNA constructs in AAV1 and a combination of at least 3 constructs in a single vector [39]. In fact, multi-skipping was also demonstrated in the GRMD model for which the skipping of at least two exons is required to restore the reading frame. Studies demonstrated efficient dystrophin rescue and muscle function improvement: following IM injection and forelimb perfusion of AAV1 [36]; percutaneous transendocardial delivery of recombinant AAV6 [35]; and AAV8 injected by locoregional transvenous perfusion of the forelimb [37]. Altogether these studies highlight the advantage of an AAV-snRNA based approach, however a 5-year follow-up study in the GRMD model revealed the progressive loss of both AAV vectors and the number of corrected muscle fibers. This effect might be due to the persistence of the dystrophic process, similar to BMD phenotypes [36]. This important finding also reported in the dKO mouse model suggests a need for repeated administration of AAV [102]. Unfortunately, the production of neutralizing antibodies following the first injection of AAV prohibits repeated injections of this type of vector, which currently represents the main challenge to the clinical application of this approach.

AAV vectors display significant advantages over other types of viral vectors: they are well tolerated after in vivo delivery; present broad in vivo biodistribution; lower capsid immunogenicity when compared to adenoviral vectors; and the availability of numerous serotypes with different tissue tropisms [113] is advantageous in treating multiple pathologies. In addition AAV-purification methods are evolving, resulting in higher yields and better transduction capacity [114]. One of the famously known limitations of AAV vectors is their low packaging capacity, however as already mentioned this is not an issue with the snRNA system given the small size of the cassette. As discussed one can even insert multiple snRNA cassettes in one vector, thereby enlarging the potential targets for example several exons or even several genes.

## 5. Conclusions

Advances in the field of genetic research led to the discovery of an increasing number of diseases found to be caused by alternative or aberrant splicing that could benefit from antisense therapy. Treatments with synthetic antisense oligonucleotides presents several challenges including low cellular uptake, low specificity when targeting specific tissues and rapid elimination from the circulation. To overcome these challenges, the vectorisation of antisense sequences using snRNA systems in viral vectors brings new possibilities. The option of using snRNAs offers many advantages amongst which specific subcellular localization and possible long-term correction, as well as limiting the potential toxicity induced by life-long administration of AOs. Moreover, since snRNAs act on the natural RNA of the targeted gene, the effects are time- and tissue-specific, avoiding any ectopic expression of the ‘corrected’ gene, as opposed to classical gene therapy approaches that use strong promoters. The ability to clone several cassettes in the same vector offers the possibility of targeting several exons or even several genes at the same time. In summary despite the immunological barriers faced by the viral vectors in the environments to which they are introduced, the viral vector-snRNA-mediated antisense approach represents a very promising tool for the treatment of genetic diseases.

## Abbreviations

The following abbreviations are used in this manuscript:

2′OMe	2′-*O*-methyl
2′MOE	2′-*O*-methoxyethyl
AAV	Adeno-associated virus
AO	Antisense oligonucleotide
BMD	Becker muscular dystrophy
CFTR	Cystic fibrosis transmembrane conductance regulator
CyPA	Cyclophilin A
dKO	Double knock out
DM1	Myotonic dystrophy type 1
DMD	Duchenne muscular dystrophy
DMPK	Dystrophia myotonica protein kinase
DNA	Deoxyribonucleic acid
ESE	Exonic splicing enhancer
ESS	Exonic splicing silencer
FDA	Food and drug administration
GAA	Acid α-glucosidase
GFP	Green fluorescent protein
GRMD	Golden retriever muscular dystrophy
HD	Huntington’s disease
HDE	Histone downstream element
hDMD	Human DMD
HIV	Human immunodeficiency virus type 1
hnRNPA1	Heterogeneous nuclear ribonucleoprotein A1
IM	Intramuscular
ISE	Intronic splicing enhancers
ISS	Intronic splicing silencers
IV	Intravenous
LNA	Locked nucleic acid
LV	Lentivirus
MBNL1	Muscleblind-like protein 1
MLV	Murine leukemia viruses
PMO	Phosphorodiamidate morpholino oligomers
PNA	Peptide nucleic acid
PPMO	Peptide phosphorodiamidate morpholino oligomers
rAAV	Recombinant Adeno-associated virus
RCL	replication competent lentiviruses
RCR	replication competent retroviruses
RNA	Ribonucleic acid
RYR1	Type 1 ryanodine receptor
scAAV	Self-complementary adeno-associated virus
SMA	Spinal muscular atrophy
snRNA	Small nuclear RNA
snRNP	Small nuclear ribonucleoproteins
SR protein	Serine/arginine-rich protein
UTR	Untranslated transcribed region
VP	Viral protein
